# A comparative *ex vivo* evaluation of colonization resistance against multidrug-resistant *Enterobacterales* in fecal samples from various animal species and strategies to augment it

**DOI:** 10.3389/fmicb.2025.1695208

**Published:** 2026-01-26

**Authors:** Lisa Osbelt, Elias Eger, Timo Homeier-Bachmann, Michael Schwabe, Marie Wende, Baerbel Hammerschmidt, Andreas Vernunft, Pavaret Sivapornnukul, Meina Neumann-Schaal, Sebastian Guenther, Till Strowig, Katharina Schaufler

**Affiliations:** 1 Microbial Immune Regulation, Helmholtz Centre for Infection Research, Braunschweig, Germany; 2 German Center for Infection Research (DZIF), Partner Site Hannover-Braunschweig, Braunschweig, Germany; 3 Epidemiology and Ecology of Antimicrobial Resistance, Helmholtz Institute for One Health, Helmholtz Centre for Infection Research, Greifswald, Germany; 4 Institute of Epidemiology, Friedrich-Loeffler-Institute Federal Research Institute for Animal Health, Greifswald, Germany; 5 Department of Experimental Animal Facilities and Biorisk Management, Friedrich-Loeffler-Institute Federal Research Institute for Animal Health, Greifswald, Germany; 6 Research Institute for Farm Animal Biology (FBN), Dummerstorf, Germany; 7 Department of Metabolomics and Services, Leibniz Institute DSMZ - German Collection of Microorganisms and Cell Cultures, Braunschweig, Germany; 8 Braunschweig Integrated Center of Systems Biology (BRICS), TU Braunschweig, Braunschweig, Germany; 9 Pharmaceutical Biology Institute of Pharmacy, University of Greifswald, Greifswald, Germany; 10 Cluster of Excellence RESIST (EXC 2155), Hannover Medical School, Hannover, Germany; 11 Center for Individualized Infection Medicine (CiiM), a Joint Venture of Hannover Medical School and Helmholtz Centre for Infection Research (HZI), Hannover, Germany; 12 University Medicine Greifswald, Greifswald, Germany

**Keywords:** *Escherichia coli*, *Klebsiella pneumoniae*, livestock, MDR pathogens, One Health, prebiotics, probiotic candidate, wildlife

## Abstract

**Introduction:**

Multidrug-resistant (MDR) pathogens, particularly *Escherichia coli* and *Klebsiella pneumoniae*, are increasingly prevalent across human, veterinary, wildlife, and environmental compartments. Although microbiota-based interventions may help limit the spread of MDR bacteria, their application beyond preclinical mouse models remains limited. Colonization resistance (CR) in complex host-associated microbiota, especially in livestock and wildlife, remains poorly understood.

**Methods:**

We investigated *in situ* CR against MDR *Enterobacterales* in fecal samples from livestock, wildlife, and humans. Using culture-dependent assays, we assessed the inhibitory capacity of native microbiota, a probiotic candidate, and selected prebiotics against MDR *E. coli* and *K. pneumoniae*. Microbial community composition and metabolic profiles were characterized using culture-independent methods, including diversity analyses and quantification of short-chain fatty acids.

**Results:**

CR varied markedly among host groups, with distinct patterns observed across wildlife, livestock, and human samples. The probiotic candidate consistently inhibited MDR *E. coli* and *K. pneumoniae* from diverse host sources, whereas prebiotics showed limited effects. Enhanced natural CR and probiotic efficacy were associated with higher microbial diversity, lower baseline abundance of endogenous *Enterobacterales*, and increased total concentrations of short-chain fatty acids.

**Discussion:**

These findings demonstrate that host-associated microbiota differ substantially in their ability to resist MDR *Enterobacterales* and that probiotic-mediated inhibition is influenced by microbial and metabolic context. Within a One Health framework, our results highlight the potential of prophylactic probiotic strategies to reduce the spread of MDR pathogens, particularly in livestock settings.

## Introduction

Antimicrobial resistance (AMR) is a critical global health challenge, resulting in a diminishing number of effective therapeutic options. The rise of multidrug-resistant (MDR) pathogens, such as extended-spectrum β-lactamase (ESBL)-producing *Escherichia coli* (EC) and carbapenem-resistant *Klebsiella pneumoniae* (KP), poses a significant threat to both human and veterinary medicine (Antimicrobial Resistance Collaborators, 2022). Compounding this problem is the spread of MDR ECs and KPs beyond clinical settings, affecting not only commercial animal husbandry and the food chain (Eger et al., 2022; Peng et al., 2022) but also profoundly impacting environmental health and wildlife (Homeier-Bachmann et al., 2021; Plaza-Rodríguez et al., 2021; Brendecke et al., 2022; Eger et al., 2024). Environmental contamination, often resulting from the improper disposal of pharmaceutical residues and wastewater, creates hotspots for the propagation and spread of MDR strains. In addition, the interconnectedness of ecosystems facilitates the transmission of these resistant bacteria to wildlife, exacerbating the challenge of AMR. However, the free movement of wild animals and their spatial separation from human activities limit our understanding of MDR EC and KP dynamics and the factors that determine the colonization of wildlife microbiota with MDR bacteria.

The microbiota plays a fundamental role in the induction, formation, and function of the host immune system (Belkaid and Hand, 2014). This symbiotic relationship has been elucidated through *in vivo* transfer experiments of defined bacterial consortia into mice, leading to mechanistic insights into microbiota–host interactions (Ivanov et al., 2009; Fischbach and Sonnenburg, 2011; Atarashi et al., 2015). Moreover, the gut microbiota serves as a frontline defense against invading MDR pathogens. These protective mechanisms, collectively referred to as colonization resistance (CR), include competition for essential nutrients (Kamada et al., 2012; Ng et al., 2013; Osbelt et al., 2021), metabolites, and environmental niches (Lam and Monack, 2014; Fang et al., 2018), as well as the production of inhibitory or toxic compounds such as short-chain fatty acids (SCFAs) (Sorbara et al., 2018; Osbelt et al., 2020).

To reduce the burden of AMR, modulators of intestinal microbial communities to increase CR have received much attention, and several novel therapeutic modalities are being developed. Among the various ways to influence CR, prebiotics (i.e., substrates that are selectively utilized by host microorganisms conferring a health benefit) and probiotics (i.e., live microorganisms that, when administered in sufficient quantities, confer a health benefit on the host) are of particular interest. However, while many studies have investigated various pre- and probiotic interventions in humans (Oliveira et al., 2020; Eberl et al., 2021; Osbelt et al., 2021; Wende et al., 2025), little is known about whether the microbiome of animals can influence CR to MDR ECs and KPs.

This study aimed to systematically investigate (i) the natural CR of fresh fecal samples from domestic pigs, dairy cattle, and their wild counterparts and (ii) the factors that influence or can influence CR. The latter particularly focused on quantification of endogenous *Enterobacterales* and SCFAs, pH determination, and analysis of microbiota on metagenomic and metatranscriptomic levels. In addition, we investigated how CR of animal samples can be influenced by the addition of β-glucan-rich algae extracts and tannic acid, both already acknowledged for their prebiotic potential, or the probiotic candidate *K. oxytoca* MK01.

## Materials and methods

### Bacterial strains and sample collection

The bacterial strains used in this study are summarized in Table 1. Each strain was preserved at −80 °C in LB broth (Carl Roth, Karlsruhe, Germany) supplemented with 20% (*v*/*v*) glycerol (anhydrous; Merck, Darmstadt, Germany). Before use, a minute of cryoculture was plated on LB agar and incubated overnight at 37 °C. One single colony was then inoculated into 5 mL of LB and grown overnight at 37 °C with vigorous shaking (130 rpm).

**Table 1:** Overview of the bacterial strains used in this study.

**Strain**	**Alternative designation**	**Species**	**Relevant characteristics or genotype**	**References**
EC-ST131	PBIO729	Eco	*bla*_CTX − M−15_, *bla*_TEM − 1_, *bla*_OXA − 1_, *tet*(AR), *aadA, aac(6′)-ib-cr*	Schaufler et al., 2013
EC-ST648	PBIO730	Eco	*bla*_CTX − M−15_, *tet*(AR), *sul1,2, strAB, aadA, aac(3)-II, mph(A), mrx, mphR*	Schaufler et al., 2013
KP-ST307	PBIO2009	Kpn	*bla*_CTX − M−15_, *bla*_TEM − 1B_, *bla*_OXA − 1_, *aac(3)-IIa, strAB, oqxAB, tet*(A), *catB3, sul2, dfrA14*	Schaufler et al., 2018
KP-ST395	YA21621	Kpn	*bla*_CTX − M−15_, *bla*_TEM − 1B_, *bla*_NDM − 1_, *bla*_OXA − 1_, *bla*_SHV − 11_, *aac(6′)-Ib-cr, aac(6′)-Ib-cr, aadA2, catA1, catB4, fosA, oqxA, oqxB, rmtC, sul1*	Osbelt et al., 2021
MK01		Kox	Probiotic candidate, *bla*_OXY−2−6_	Osbelt et al., 2021

Eco, *Escherichia coli*; Kox, *Klebsiella oxytoca*; Kpn, *Klebsiella pneumoniae*; ST, sequence type.

During 2021, we collected 82 fecal samples of various animal origins from different locations. Wildlife fecal samples were collected from wild boar (*n* = 14) and fallow deer (*n* = 15) in geographically distinct locations across Western Pomerania, Germany (Supplementary Table S1). Livestock-associated fecal samples were collected from farms in Western Pomerania and one farm in Schleswig-Holstein (Preetz; dairy cattle, *n* = 5). In Western Pomerania, samples were collected from an organic farm in Dummerstorf (domestic pigs, *n* = 24; dairy cattle, *n* = 15), Riems (domestic pigs, *n* = 5), and a conventional farm in Ivenack (dairy cattle, *n* = 4).

Fresh fecal samples from wild boar and fallow deer were collected within a maximum of 3–5 h of defecation from natural habitats to minimize changes in microbiota and volatile metabolites. Fecal samples from domestic pigs and dairy cattle were collected immediately after defecation. All animal samples were transferred into sterile containers and stored on ice during transport to the laboratory to minimize bias and degradation (Jaramillo-Jaramillo et al., 2024). Metadata available animal samples are listed in Supplementary Table S1.

### Human data collection

During 2021, we collected nine fecal samples from healthy adult volunteers from the local area of Braunschweig (Germany) as controls for the animal study. Human sample and data collections for healthy participants have been performed in agreement with the guidelines of the Helmholtz Center for Infection Research, Braunschweig, Germany, the Ethics Committee Lower Saxony (permit No. 8629_BO_K_2019), and the European Data Protection Laws (Europäische Datenschutz-Grundverordnung DSGVO). Patients or patient characteristics (sex, gender, ethnicity) were not part of the analysis and no patient data are reported. All human donors declared themselves as healthy (no antibiotics intake within the last 3 month, no chronic diseases). Human samples were self-collected by volunteers under standardized instructions, with participants advised to send fresh fecal samples to the laboratory within 24 h of defecation. Upon arrival, all samples were immediately aliquoted and snap-frozen at −80 °C until further analysis; a portion of each sample, however, was immediately used for characterization and determination of CR. All human donors have signed a letter of informed consent in accordance with the World Medical Association Declaration of Helsinki (Version 2013).

### Characterization of fecal samples

Fresh, cooled and native fecal samples from wildlife, domestic animal, and humans were processed as follows: For SCFA analysis and 16S rRNA gene sequencing, 50–100 mg aliquots were immediately frozen at −80 °C, as described previously (Osbelt et al., 2020). Another aliquot of 20–100 mg sample was diluted in 1 mL of sterile saline solution to assess the pH, and 100 μL of resuspended fecal sample was plated on chromogenic agar plates (CHROMagar orientation, Mast Diagnostica, Reinfeld, Germany). Following a 24-h incubation at 37 °C, the abundance and diversity of the commensal enterobacteria and facultative anaerobes were assessed based on colony color and morphology on chromogenic agar. Downstream correlation analysis using Spearman's rank correlation was performed for all samples to evaluate relationships between SCFA concentrations (and fecal DNA content) and bacterial CFU counts. Multiple comparisons were corrected using the Benjamini–Hochberg procedure, and correlations with an adjusted FDR < 0.05 were considered statistically significant.

### Quantification of colonization resistance against MDR bacteria

To determine the CR, an aliquot of each individual fecal sample was inoculated with each of the four MDR bacteria as previously described (Osbelt et al., 2021). Each fecal sample-pathogen combination was tested in a single, independent biological replicate due to logistical constraints in acquiring diverse fresh samples. Briefly, the overnight cultures of the MDR bacteria were diluted 1:100 in 5 mL of fresh LB and incubated at 37 °C with agitation until the bacterial cultures reached an OD_600_ = 0.2. The fecal sample was resuspended in 1 mL of sterile PBS and homogenized by bead beating with 1 mm zirconia beads (Carl Roth, Karlsruhe, Germany) two times for 25 s each using a Mini-Beadbeater-96 (BioSpec, Bartlesville, OK, USA) and a FastPrep system (MP Biomedicals, Eschwege, Germany). Next, the resuspended fecal samples were inoculated with 10 μL of an adjusted bacterial culture, corresponding to an inoculum of 8 · 10^5^-2 · 10^6^ CFU. The samples were immediately transferred to an anaerobic jar and incubated at 37 °C for 24 h under microaerophilic conditions. The next day, viable amounts of the corresponding MDR strains were recovered on LB agar supplemented with 4/2/16 μg/mL cefotaxime/ciprofloxacin/tetracycline (EC-ST131, EC-ST648, and KP-ST307) or 25 μg/mL chloramphenicol (KP-ST395) via serial dilutions. To ensure that recovered colonies corresponded exclusively to the inoculated MDR *Enterobacterales* strains and not potential indigenous resistant strains, the colonies were confirmed visually by colony morphology. In accordance with our previous study (Osbelt et al., 2021), samples that were able to maintain the inoculum level or even inhibit further growth were designated “resistant: RES”. If more than 100-fold outgrowth was observed (threshold = 1 · 10^10^ CFU), the samples were considered “susceptible = SUS”. All samples in between were considered “intermediate = INT”.

### Analysis of short-chain fatty acid concentrations

The SCFA content was quantified via a previously described method (Neumann-Schaal et al., 2015), with slight modifications. Approximately 50–100 mg of each fecal sample was weighed and stored at−80 °C until further processing. For SCFA extraction, samples were resuspended in 600 μL of water spiked with an internal standard (30 μL of *o*-cresol/mg sample) and homogenized. Then, 400 μL of the mixture was acidified with 50 μL of 65% HPLC-grade sulfuric acid and mixed vigorously for 5 min. The mixture was extracted with 200 μL of *tert*-butyl methyl ether, and the ether phase was analyzed via GC–MS via an Agilent GC–MSD system (7890B coupled with 5977 GC) equipped with a high-efficiency source (HES), a quadrupol mass spectrometer, and a Gerstel RTC system. Chromatography was performed on an Agilent VF-WAX-MS column (30 m length, 0.25 mm inner diameter; Agilent, Santa Clara, CA, USA) with a constant flow of 1 mL/min helium. The temperature program was as follows: 55 °C for 1 min, ramp from 10 °C min to 250 °C for 2 min. The solvent delay time was 2.4 min. To convert retention times to retention indices, a retention index marker (i.e., *n*-alkanes with *n* = 10–22 in cyclohexane) was used. Quantification was performed via an external calibration curve with authentic standards. Data analysis was performed as previously described (Neumann-Schaal et al., 2015; Hofmann et al., 2018).

### Probiotic and prebiotic intervention studies

The well-characterized *K. oxytoca* strain MK01 was used (Osbelt et al., 2021) to investigate the influence of a probiotic strain on the CR of MDR bacteria. Both the respective MDR and probiotic strains grown to the exponential phase (i.e., OD_600_ = 0.2) were added to the fecal suspension. The strains were added at a ratio of 1:10 (MDR:MK01). The further procedure was carried out according to the CR determination.

To study the effects of natural compounds with prebiotic potential on the CR of fecal samples from dairy cattle and domestic pigs (*n* = 10 each), we used tannic acid (Sigma-Aldrich, St. Louis, MO, USA) and an in-house prepared β-glucan-rich algal extract. For experimental application, 200 μg/mL tannic acid or 400 μg/mL β-glucan-rich algal extract was prepared. To ensure precise dosing, the prebiotics were initially dissolved in water to the appropriate concentrations, then aliquoted into individual tubes and lyophilized to yield exact final masses of 400 μg of algae biomass and 200 μg of tannic acid per tube. Before use, the prebiotics were reconstituted in 1 mL of BHI (Merck, Darmstadt, Germany), and further procedures were carried out according to the determination of CR as described previously. The CR was inversely proportional to the CFU/g of the MDR strain.

### DNA extraction and 16S rRNA gene sequencing of fecal samples

Microbial DNA from fecal samples was isolated and purified on a magnetic bead basis via the Zymo MagKit (Zymo Research, Freiburg, Germany) according to the manufacturer's instructions. Briefly, 100 mg of fecal sample was mixed with 700 μL of lysis buffer and 100 μL of zirconia beads (0.1 mm diameter; Carl Roth, Karlsruhe, Germany). Bacterial lysis was performed by mechanical disruption using a Mini-BeadBeater-96 three times for 5 min with 5 min of incubation on ice in between. After centrifugation, 200 μL of the supernatant was transferred to a 96-deep well plate (Nunc; Thermo Fisher Scientific, Waltham, MA, USA), and 600 μL of Magbinding Buffer was added. Then, 40 μL of MagBinding beads were added, and the following washing steps were performed on a Tecan Fluent (Tecan, Männedorf, Switzerland).

16S rRNA gene amplification of the V4 region (F515/R806) was performed according to an established protocol previously described (Caporaso et al., 2011). Briefly, DNA was normalized to 25 ng/μL and used for sequencing PCR with unique 12-base Golary barcodes incorporated via specific primers (purchased from Sigma–Aldrich, Burlington, MA, USA). PCR was performed via Q5 polymerase (New England Biolabs, Ipswich, MA, USA) in triplicate for each sample, using PCR conditions of initial denaturation for 30 s at 98 °C, followed by 25 cycles (10 s at 98 °C, 20 s at 55 °C, and 20 s at 72 °C). After pooling and normalization to 10 nM, the PCR amplicons were sequenced on an Illumina MiSeq platform (Illumina, San Diego, CA, USA) via 300 bp paired-end sequencing (PE300). The resulting reads were assembled, filtered and clustered via the Usearch9 software package (http://www.drive5.com/usearch/). The sequences were filtered for low-quality reads and binned on the basis of sample-specific barcodes via QIIME v1.8.0 (Caporaso et al., 2010). Merging was performed via -fastq_mergepairs with fastq_maxdiffs 30. Quality filtering was performed via fastq_filter (-fastq_maxee 1) with a minimum read length of 300 bp and a minimum number of reads per sample = 1,000. The reads were clustered into 97% ID OTUs via open reference OTU picking, and representative sequences were determined via the UPARSE algorithm v7.1 (Edgar, 2010). Abundance filtering (OTU clustering > 0.5%) and taxonomic classification were performed via the RDP Classifier running at the 80% bootstrap confidence cutoff (Wang et al., 2007). Sequences without matching reference data were assembled *de novo* via UCLUST v1.0.0. Phylogenetic relationships between OTUs were determined via PyNAST alignment via FastTree v2.2 (Price et al., 2010). The resulting OTU absolute abundance table and mapping file were used for statistical analyses and data visualization in the R statistical programming environment package phyloseq v4.5.0 (McMurdie and Holmes, 2013). The entire One Health dataset was analyzed accordingly and compared for species richness, diversity and microbiota composition (two samples were excluded because of poor DNA quality).

### RNA sequencing and metatranscriptomic analysis

To analyze the taxonomic changes in the fecal microbiota and transcriptomic adaptations of EC-ST131 and EC-ST648 to their respective microbiome environments, we extracted and purified RNA from ten selected fecal samples of domestic pigs (*n* = 6) and wild boars (*n* = 4) challenged in the *ex vivo* screening system described above. After anaerobic incubation at 37 °C for 24 h, transcriptomic activity was immediately stopped by cooling in liquid nitrogen for 3–4 s. Samples were mechanically disrupted via the FastPrep system. RNA purification and enrichment were performed according to the manufacturer's instructions. The RNA was checked for purity and quantified fluorometrically via a Qubit 4 fluorometer (Thermo Fisher Scientific, Waltham, MA, USA). Total RNA was sent to the Competence Center for Genomic Analysis (CCGA, Kiel, Germany) on dry ice, and after rRNA depletion and mRNA library preparation, it was paired-end sequenced (2 x 100 bp reads) on an Illumina NovaSeq 6000 platform (Illumina, San Diego, CA, USA).

The raw reads were adapter and quality trimmed via Trim Galore v. 0.6.7 (https://github.com/FelixKrueger/TrimGalore). To remove the remaining rRNA sequences, the trimmed reads were aligned to the SILVA LSU and SSU database v. 138 (Quast et al., 2012) via Bowtie 2 v. 2.4.5 (Langmead et al., 2018). Only the unmapped paired reads were used for further analysis. The paired reads were taxonomically classified via Kraken 2 (v. 2.1.3) with the prebuilt PlusPF database v. 2024/01/12. On the basis of the Kraken 2 assignment, Bracken v. 2.9 was used to calculate the abundance of genera in the individual samples. Then, α diversity was assessed via KrakenTools v. 1.2 (Lu et al., 2022).

The genomes of EC-ST131 (Biosample SAMEA113549584) and EC-ST648 (Biosample SAMN07765387) were annotated via Prokka v. 1.14.6 (Seemann, 2014). Next, the unmapped paired reads were mapped with Bowtie 2 (model: –very-sensitive-local) using each of the assembled genomes of EC-ST131 and EC-ST648 as references. The gene counts were calculated via featureCounts v. 2.0.1 (Liao et al., 2013) on the basis of the annotations of EC-ST131 and EC-ST648. Differentially expressed genes were called via DESeq2 v. 1.44.0 (Love et al., 2014) in default mode (threshold, |log2FC| ≥ 2 and *p* ≤ 0.05). All differentially expressed genes were assigned to the COG (Clusters of Orthologous Groups of proteins) categories via eggNOG-mapper v. 2.0.1 (Cantalapiedra et al., 2021).

### Statistical analysis

The collection of human fecal samples was randomized and blinded. No other data collection or analysis was performed in a blinded manner to the conditions of the experiments. No statistical methods were used to predetermine sample sizes, but our sample sizes are similar to those reported in previous publications (Osbelt et al., 2021; Wende et al., 2025). No animals or data points were excluded from the analyses. P values were analyzed via Wilcoxon matched rank test, Kruskal–Wallis test (for two-group comparisons), or one-way ANOVA (multiple groups) with Tukey's *post hoc* test, as indicated in each figure legend. No specific adjustments were made for multiple comparisons, as the applied *post hoc* tests specifically account for multiple comparisons and maintain α at the specified level (0.05). For 16S plot analysis, OTUs with a Kruskal–Wallis test score < 0.05 were considered for analysis. *P-*values lower than 0.05 were considered significant: ^*^p < 0.05; ^**^p < 0.01; ^***^p < 0.001; ^****^p < 0.0001. Descriptions of each statistical test and exact *p* values are provided in the source data tables for each figure item.

## Results

### The natural level of resistance to various multidrug-resistant bacteria is highly variable in different animal hosts

**Figure 1:**
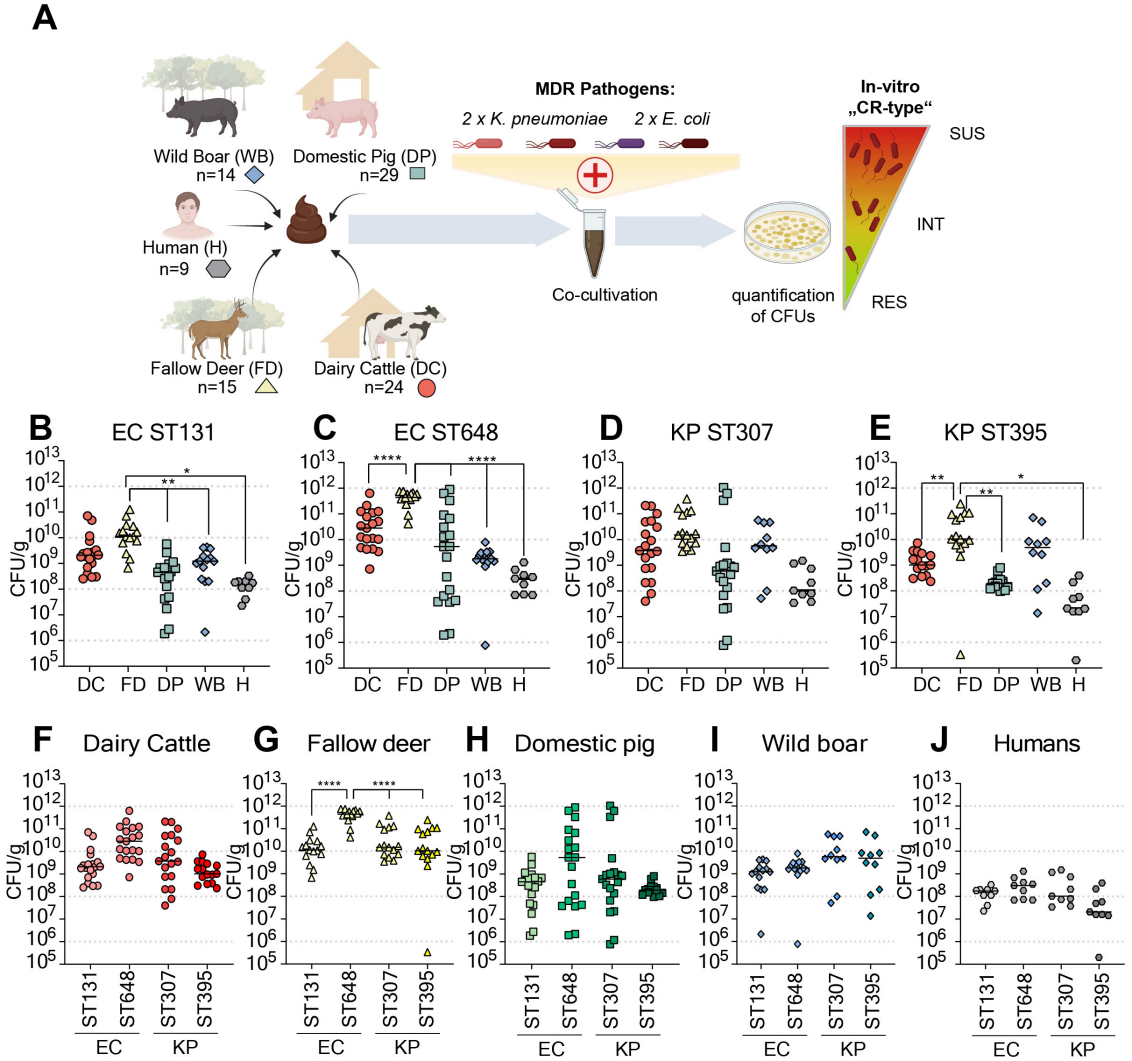
**(A)** Schematic of the *ex vivo* screening system assay to assess the level of colonization resistance. Fecal samples from various hosts, wildlife, livestock, and humans were deliberately inoculated with different strains of multidrug-resistant (MDR) *Escherichia coli* (EC) and *Klebsiella pneumoniae* (KP) representative of high-risk sequence types (STs). Following inoculation, the samples were incubated under anaerobic conditions for 24 h, after which the MDR bacterial loads were quantified via selective agar plates. **(B–E)** Quantification of colony forming units (CFUs) per gram of feces for ESBL-producing *E. coli* belonging to strains ST131 **(B)** and ST648 **(C)** and carbapenem-resistant *K. pneumoniae* strains ST307 **(D)** and ST395 **(E)**. **(F–J)** Comparative analysis of CFUs per gram of feces for MDR bacteria in different host groups, including ruminant [dairy cattle (DC), **(F)**; fallow deer (FD), **(G)**], swine [domestic pig (DP), **(H)**; wild boar (WB), **(I)**], and human (H) samples **(J)**. Statistical significance was determined by ordinary one-way analysis of variance (ANOVA) and Tukey's multiple comparison test, with significance levels indicated as follows: ^*^p < 0.05; ^**^p < 0.01; ^***^p < 0.001; ^****^p < 0.0001.

To characterize natural CR levels across various animal hosts (Supplementary Table S1), including wild boar, fallow deer, domestic pig, and dairy cattle, in comparison with a limited number of humans, we applied a simplified *ex vivo* screening system (Figure 1A), previously developed for assessing CR levels in humans (Osbelt et al., 2021). For analysis of CR, the well-characterized ESBL-producing *E. coli* strains EC-ST131 (PBIO729) and EC-ST648 (PBIO730), as well as the carbapenem-resistant *K. pneumoniae* strains KP-ST307 (PBIO2009) and KP-ST395 (YA21621), were selected. The MDR EC strains were isolated from a urinary tract infection of a dog and the feces of a wild bird (Schaufler et al., 2013). KP-ST307 originated from a rectal swab of a Guinean rat (Schaufler et al., 2018), and KP-ST395 was isolated from a hospitalized patient at the University Hospital Magdeburg (Magdeburg, Germany) (Osbelt et al., 2021). In this screening method, a high CR corresponds to low CFU/g values of the respective MDR strain, whereas a low CR is indicated by high CFU/g values.

The CR to the ESBL-producer EC-ST648 was lower than that to EC-ST131, except in the wild boar samples, which had similar CRs to both strains (Figures 1B, C). The difference in CR to the KP strains between the different animal origins was much lower than that for the EC strains (Figures 1D, E). In general, the fecal samples from fallow deer presented the lowest CR to the EC and KP strains. When comparing the CR values of the different hosts against the MDR strains, we observed the most significant variability in samples from domestic pigs, spanning seven orders of magnitude from the most protected to the most susceptible sample (Figure 1H). In addition, samples from fallow deer had the lowest CR against all MDR strains among the hosts tested (Figures 1F–J). Overall, we recovered, on average, fewer CFUs from the human control samples than from the animal samples, although this could be an artifact due to the small sample size and high variability that was observed in earlier studies with KP-ST395 (Osbelt et al., 2021).

### The abundance and diversity of *Enterobacterales* and pH levels significantly varied across different host species

**Figure 2:**
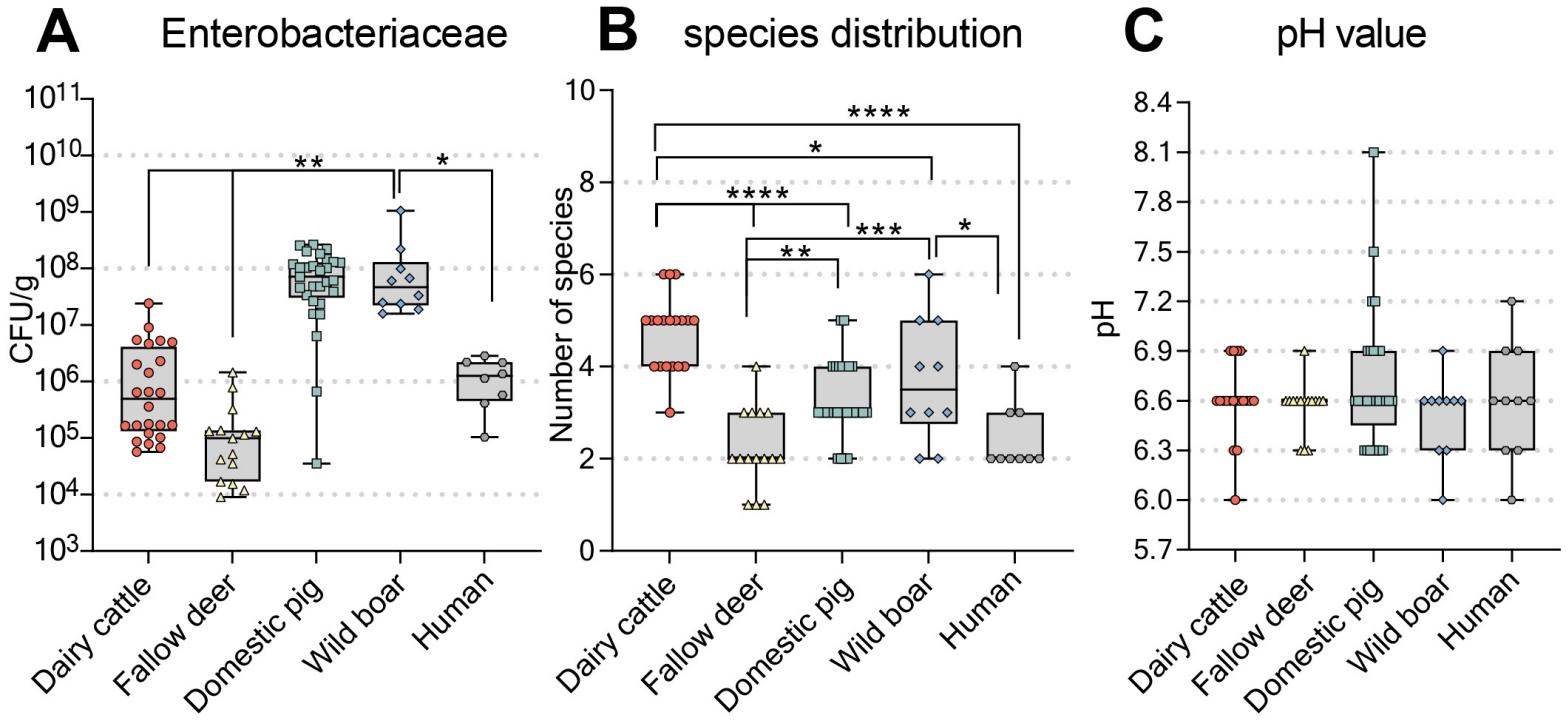
Overview of the characteristics of fecal samples from different animal origins. **(A)** Quantification of *Enterobacterales* content expressed as CFU per gram of fecal sample. **(B)** Assessment of superficial species diversity by staining for different bacterial species on chromogenic agar plates. **(C)** Measurement of pH in resuspended fecal samples. The median is represented by the line in the box, while the boxplot represents the interquartile range (25th to 75th percentile) of the dataset. Whiskers extend to the minimum and maximum values. Statistical significance was determined by ordinary one-way ANOVA and Tukey's multiple comparison test, with significance levels indicated as follows: ^*^p < 0.05; ^**^p < 0.01; ^***^p < 0.001; ^****^p < 0.0001.

To evaluate the factors that could explain the observed differences in natural CR values between the different hosts, we first quantified the influencing factors identified in previous studies, such as the abundance of endogenous *Enterobacterales* (Velazquez et al., 2019; Eberl et al., 2021; Osbelt et al., 2021) and the pH value (Sorbara et al., 2018; Osbelt et al., 2020). The results are summarized in Figure 2.

The levels of endogenous *Enterobacterales* were highest in samples from domestic pigs (median, 7.19 · 10^7^ CFU/g) and wild boars (median, 2.92 · 10^7^ CFU/g) and lower in dairy cattle (median, 1.74 · 10^5^ CFU/g) and fallow deer (median, 9.91 · 10^4^ CFU/g; Figure 2A). In contrast, samples from dairy cattle had the highest species diversity (median, 5), whereas fecal samples from domestic pigs (median, 3) and wild boars (median, 3.5) were less diverse (Figure 2B). Interestingly, correlating with the lowest level of CR in the *ex vivo* screening system, the fallow deer samples presented the lowest level of *Enterobacterales* and species diversity (median, 2; Figures 2A, B). The median pH values of the fecal samples were similar across all the animals and humans (median, 6.6; Figure 2C). To exclude any impact of the input of fecal material on the outcome of the spike-in assay, we performed a correlation analysis of DNA concentration and measured CFUs (Supplementary Table S2), which did not display a significant correlation except for CFUs of KP-ST395 which negatively correlated with measured DNA concentration in domestic pig samples. As this was the only significant correlation found, we assumed a rather limited impact of the overall bacterial load on the outcome of the ex vivo assay.

### Short-chain fatty acid composition varied widely among the fecal samples and significantly correlated with suppression of *K. pneumoniae* in pigs

Since we detected significantly higher CRs in fecal samples from pigs, especially domestic pigs, we considered a different diet composition as a possible explanation. Diet composition changes the production of SCFAs, which are known to influence CR (Jacobson et al., 2018; Osbelt et al., 2020). Given that SCFA production—shaped by dietary composition—affects CR, we considered SCFA-mediated dietary differences as a potential explanation for the elevated CRs observed in pig fecal samples, especially those from domestic pigs.

**Figure 3:**
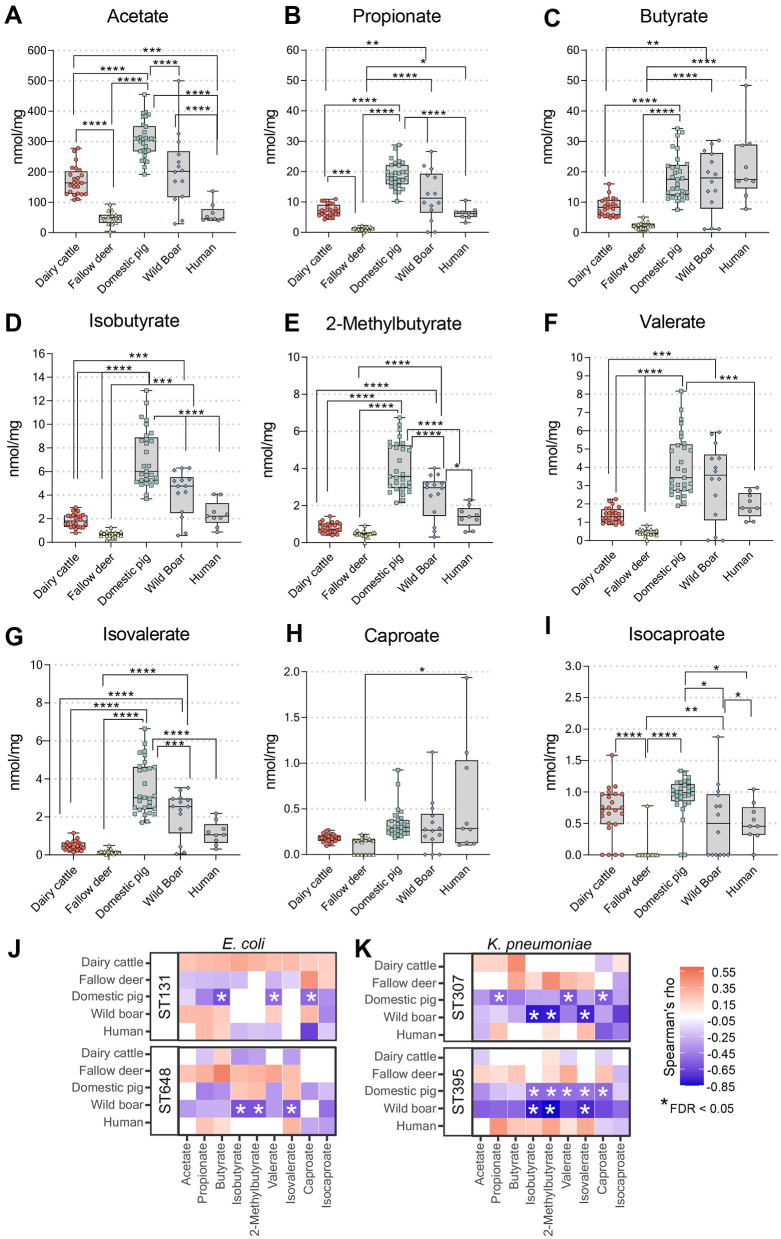
Quantification of short-chain fatty acids (SCFAs) in fecal samples from various origins. **(A–I)** SCFA levels in individual animal and human samples, expressed in nmol/mg. Each data point represents the mean of two replicates for each sample. The median is represented by the line in the box, while the boxplot represents the interquartile range (25th to 75th percentile) of the dataset. Whiskers extend to the minimum and maximum values. Statistical significance was determined by ordinary one-way ANOVA and Tukey's multiple comparison test, with significance levels indicated as follows: ^*^p < 0.05; ^**^p < 0.01; ^***^p < 0.001; ^****^p < 0. 0001. **(J, K)** The association between SCFAs and colonization resistance (CR) was assessed using Spearman's rank correlation analysis. Significant negative correlations (FDR < 0.05) were observed between several SCFAs and colony-forming unit (CFU) counts per gram of *E. coli* (ST131 and ST648) and *K. pneumoniae* (ST307 and ST395) across different fecal origins.

Overall, we detected significantly higher average levels of all SCFAs in fecal samples from pigs, especially domestic pigs, which correlated with the highest level of protection against various MDR bacteria in our *ex vivo* screening system (Figure 3). In general, domestic animals had higher levels of SCFAs in their feces than did their wild counterparts. Compared with the wild boar samples, the domestic pig samples contained significantly higher levels of acetate, propionate, 2-methylbutyrate, isobutyrate, isovalerate, and isocaproate. The ruminant samples differed significantly in their acetate, propionate, butyrate, valerate, isobutyrate, and isocaproate levels. However, we observed notable intraindividual differences among individual animals, with the greatest variability in samples from domestic pigs and wild boars. On average, human samples, as well as samples from wild ruminants and dairy cows, had comparable levels of the abundant SCFAs acetate, propionate, and valerate. However, compared with those in pigs (domestic and wild), the levels were rather low. For butyrate, values were similar across ruminants (domestic and wild), but levels in the human samples were similar to those in pigs. For branched-chain SCFAs, we also detected higher levels in porcine samples than in human and ruminant samples.

A more detailed analysis stratifying all samples by SCFA composition and CFU levels in the CR spike-in assay revealed that suppression of KP-ST395 and KP-ST307 was negatively correlated with concentrations of propionate, butyrate, 2-methylbutyrate, isobutyrate, valerate, and caproate in domestic pig samples (Figures 3J, K). In wild boar samples, suppression correlated negatively with levels of 2-methylbutyrate, isobutyrate, and isovalerate, while no significant correlation was found for the ruminant samples with specific SCFA. In addition, domestic pig samples showed significant negative correlation with Butyrate, Valerate, and Caproate for EC-ST131 as well as wild boar samples showed significant negative correlation with Isobutyrate, 2-Methylbutyrate and Isovalerate for EC-ST684. Thus, specific SCFAs, mainly butyrate and propionate and its branched-chained variants, play a significant, though not exclusive, role in suppressing Enterobacteriales and specifically *K. pneumoniae* strains.

### Microbiome profiling revealed less species diversity in livestock than in wildlife

**Figure 4:**
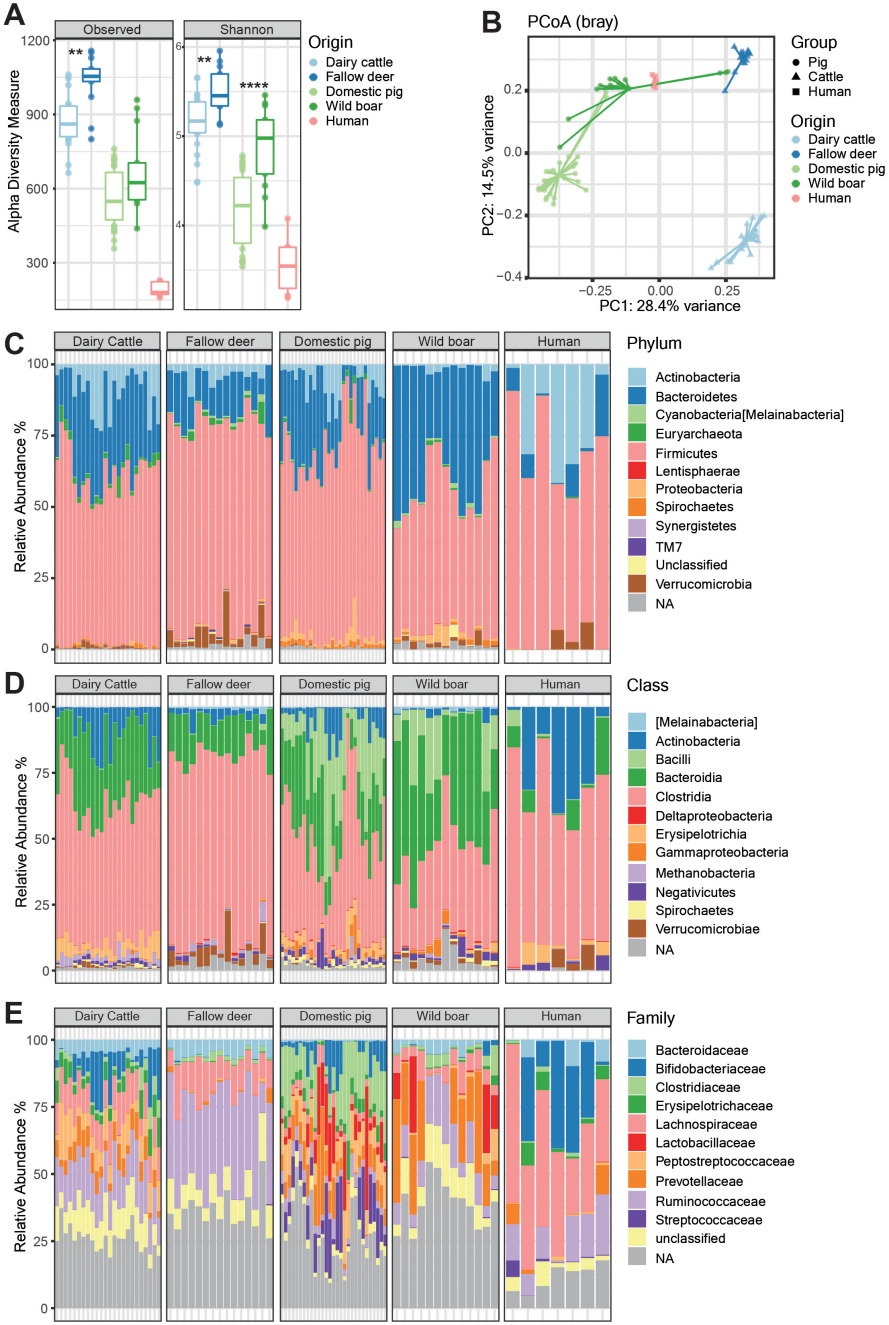
Microbiota analysis using 16S rRNA gene sequencing of fecal samples from different origins. **(A)** Alpha diversity displayed as observed species (left) and the Shannon diversity index (right) among different animal groups (dairy cattle, fallow deer, domestic pigs, and wild boars) and humans. Statistical significance was determined via the Kruskal–Wallis test, with ^**^p < 0.01 and ^****^p < 0.0001. **(B)** Principal coordinate analysis (PCoA) plot of beta diversity among the four sample groups. **(C–E)** Composition of the fecal microbiota in the four different animal groups and humans shown at the phylum, class, and family level.

The microbiome analysis was conducted via 16S rRNA amplicon sequencing. We observed a striking contrast in alpha diversity, i.e., within-sample complexity, between wild and domesticated animals (Figure 4A). Specifically, fecal samples from wild ruminants and wild boars presented significantly more species-rich and more diverse microbiomes than their respective domesticated counterparts did, as indicated by the increased number of observed species and Shannon diversity indices, respectively (wild ruminant vs. dairy cattle, *p* < 0.01; wild boar vs. domestic pig, *p* < 0.0001). Furthermore, while α diversity varied significantly only within the wild ruminant vs. dairy cattle pair (*p* < 0.01), a discernible trend emerged: ruminants, including wild and domesticated species, harbored a microbiota of greater complexity than pigs.

The analysis of beta diversity using Bray-Curtis distances and PCoA further delineated the distinct microbial signatures of wild vs. domesticated animals, with clear separations observed between wild ruminants and dairy cattle, and between wild boars and domestic pigs (Figure 4B). Interestingly, porcine fecal samples showed greater similarity to one another, occasionally posing challenges for differentiation. Notably, human samples were more similar to those from pigs than those from dairy cattle were, suggesting that microbial characteristics are shared between humans and pigs, albeit with different clustering patterns.

Taxonomic analysis at phylum, class and family-level compositions of the different animal species revealed consistent trends within species, but individual variation persisted (Figures 4C–E). The porcine microbiota generally had relatively high abundances of Bacilli and Proteobacteria, whereas Clostridia and Bacteroidia dominated the ruminant microbiota. Compared with all animal groups, human samples, while showing interindividual variation, had fewer Bacilli and a greater prevalence of Actinobacteria. Interestingly, selected human samples were similar to wild ruminant and wild boar samples in terms of the presence of *Verrucomicrobia*.

### Microbial diversity influences the resistance to multidrug-resistant *Escherichia coli* colonization

**Figure 5:**
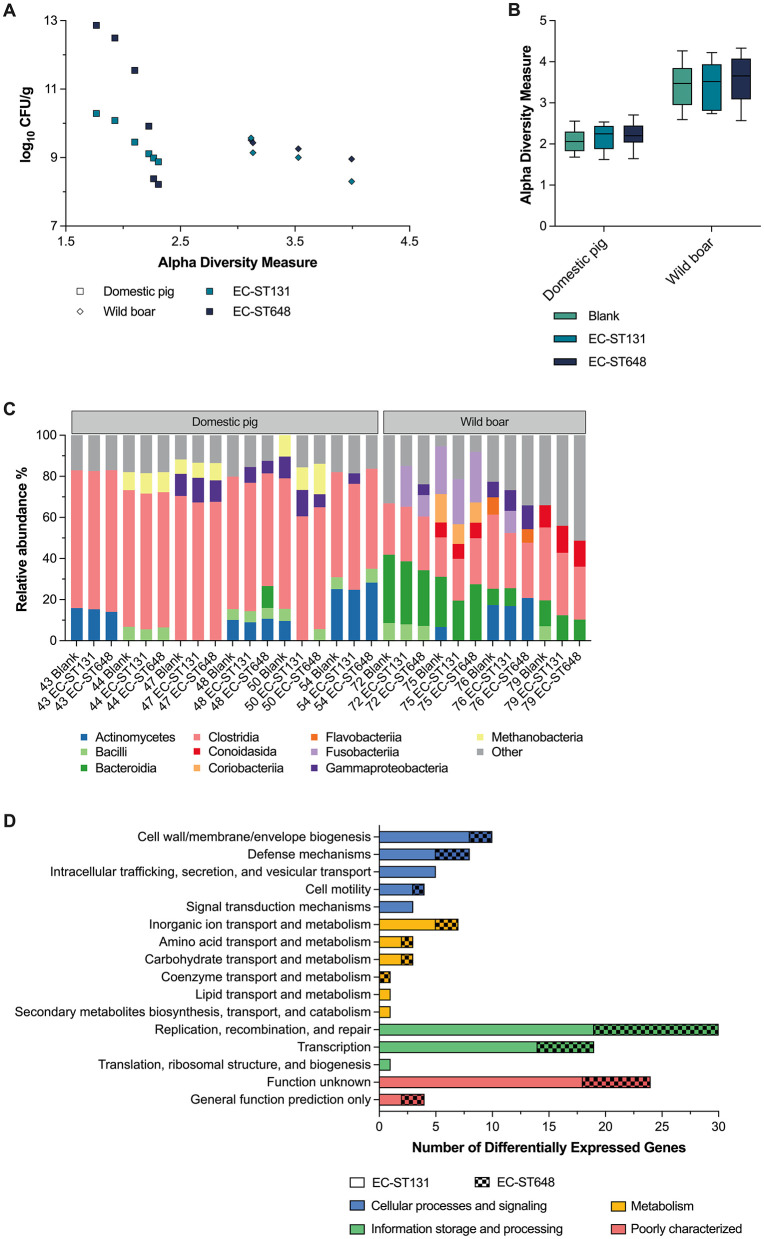
Summary of the metatranscriptome analysis of selected samples from domestic pigs and wild boars. **(A)** The plot shows a negative correlation between the Shannon diversity index and the log-transformed CFU count of the added MDR *Enterobacterales* strain. **(B)** Comparison of the Shannon diversity indices between nonspiked (blank) samples and samples spiked with MDR EC. **(C)** Taxonomic composition of nonspiked and spiked samples. **(D)** Functional classification of differentially expressed genes in the spiked samples compared to the nonspiked samples based on the Clusters of Orthologous Groups (COG) database.

To investigate changes in taxonomic composition and gene expression when our two exemplary MDR EC strains invade the microbiome, we used a metatranscriptomic approach that allows comprehensive taxonomic resolution and detection of transcriptionally active microorganisms. For this purpose, we performed a comparative analysis of selected samples from domestic pigs and wild boars, which presented the highest taxonomic diversity (Figure 5). In agreement with the results of the 16S rRNA analysis, the microbial diversity of the wild boar samples was greater than that of the domestic pig samples (Figure 5A). Furthermore, our analysis revealed a clear correlation between the CR of both MDR EC strains and the Shannon diversity index of the fecal samples. In samples from domestic pigs, the increase in Shannon diversity index was associated with a strong increase in CR, especially from CR to EC-ST648. In contrast, the CR of the wild boar samples increased gradually with increasing microbial diversity. However, when the differences in α diversity due to the presence of the MDR EC strains were compared, no significant changes were observed compared with the non-spiked fecal sample (blank; Figure 5B), indicating that the introduced MDR EC strains did not alter the overall microbial community structure in either domestic pigs or wild boars. This was confirmed by our analysis of the taxonomic composition of the spiked and non-spiked samples (Figure 5C), which revealed that the existing microbiota remained stable at the class level. This may indicate that the existing microbiota in these animals is robust enough to maintain their structure even in the presence of nonindigenous MDR EC strains.

To determine whether and how the microbial context promotes CR in MDR EC strains, we characterized the transcriptomic landscape of EC-ST131 and EC-ST648 in fecal samples (Figure 5D). Gene expression analysis comparing spiked samples with non-spiked samples revealed 134 differentially expressed EC genes, of which 95 were upregulated and 39 were downregulated, in at least eight different domestic and wild boar samples. Functional categorization using the COG (Clusters of Orthologous Groups of Proteins) database assigned the largest proportion of these genes (*n* = 50) to “information storage and processing”, specifically within the “transcription” and “replication, recombination, and repair” subcategories. Given the minor observed changes in metabolism-associated genes, this might suggest that the MDR EC strains did not significantly alter their nutrient utilization spectrum compared to the indigenous gut microbiota. However, these results should be interpreted with caution, as transcriptomic analysis may not fully capture the nuanced strain-specific activities and interactions, especially in complex microbial communities.

### Prebiotic and probiotic interventions increased colonization resistance

**Figure 6:**
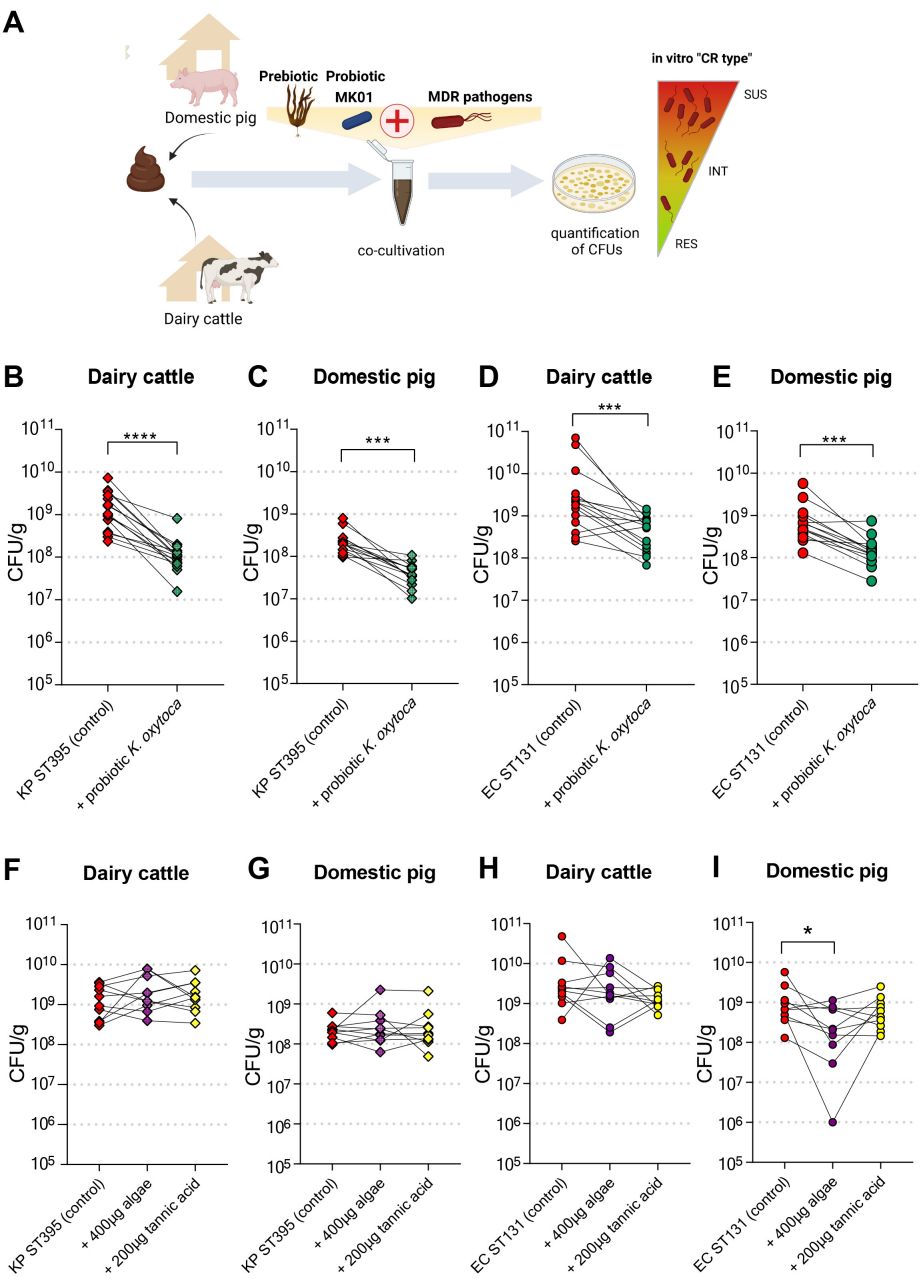
Pro- and prebiotic interventions to influence the colonization resistance of animal fecal samples. **(A)** Samples from livestock were spiked with a well-known probiotic candidate (*K. oxytoca* MK01) or treated with prebiotics (algae extract or tannic acid) to assess the impact of CR on *K. pneumoniae* ST395 (KP-ST395) or *E. coli* ST131 (EC-ST131). **(B–E)** Quantification of colony forming units (CFUs) per gram of feces from dairy cattle and domestic pigs without (red) and with (green) the probiotic *K. oxytoca* strain against KP-ST395 **(B, C)** and EC-ST131 **(D, E)**. **(F–I)** Effects of 400 μg/mL algae extract (purple) or 200 μg/mL tannic acid (yellow) in comparison with control samples without prebiotic intervention (red) in dairy cattle and domestic pig samples against KP-ST395 **(F, G)** and EC ST131 **(H, I)**. Statistical significance was determined by the Wilcoxon matched rank test, with ^*^p < 0.05; ^***^p < 0.001; ^****^p < 0.0001.

We previously demonstrated the protective properties of the human stool-derived *K. oxytoca* strain MK01 in human samples and mice (Osbelt et al., 2021). Furthermore, it is well established that prebiotic interventions can influence CR *in vitro* and *in vivo*. For example, tannins (e.g., tannic acid) and β-glucan-rich algae have shown promising properties (Schulze et al., 2021; Choi et al., 2023). Therefore, we investigated the influence of *K. oxytoca* MK01, tannic acid, and algae extract, which are probiotic and prebiotic candidates, respectively, in domestic animal samples (Figure 6A), as these interventions may provide a practical approach to improve CR against MDR pathogens in livestock. Specifically, we considered a co-inoculation ratio of 1:10 (pathogen:probiotic), selected based on previous studies in which we extensively screened the protective and non-protective capabilities of strains across a variety of assay conditions, inoculation ratios, and experimental settings (Supplementary Table S2). This ratio was supported by testing various MK01:KP-ST395 ratios in prepration of the experiments revealing a robust, tenfold reduction in pathogen burden, specifically at the 1:10 (pathogen:probiotic) inoculation ratio (Supplementary Figure 1).

Compared with those in untreated dairy cattle samples, significant reductions in the CFU of EC-ST131 and KP-ST395 were observed in dairy cattle samples after 24 h of microaerophilic incubation with the probiotic strain (Figures 6B, D). The reduction ranged from 10- to 100-fold. Interestingly, two fecal samples presented a tenfold increase in the CFU of EC-ST131 despite the presence of the probiotic *K. oxytoca* strain. In contrast, domestic pigs presented a lower average increase in CR in fecal samples than did dairy cattle (Figures 6C, E). The decrease in the CFU of MDR bacteria was only 10-fold. Interestingly, there was also a decrease in CR in one sample from a domestic pig challenged with EC-ST131. Taken together, these results confirm the results of the previous work and show that CR can be increased by the presence of the probiotic candidate MK01. However, the fact that the effect of MK01 varied between samples makes it likely that the protection is also mediated by a direct mechanism involving other commensal species that contribute to CR (Zmora et al., 2018).

In contrast, prebiotics did not affect the CR to KP-ST395 in dairy cattle (Figure 6F). In some individual samples, a decrease in CR was obtained, especially when the samples were incubated with algal biomass. The effect of CR on EC-ST131 in dairy cattle samples was more pronounced (Figure 6H). While some samples showed no change in CR, individual samples presented an increase in CR in the presence of algal biomass, and two samples presented a decrease in CR. Like KP-ST395 in dairy cattle, neither prebiotic clearly affected the CR of domestic pig samples (Figure 6G). One sample presented a decrease in the CR in the presence of algae and tannic acid of 10-fold. In domestic pig samples, the presence of algal biomass resulted in a statistically significant increase in CR to EC-ST131 (*p* < 0.05), whereas tannic acid had no effect on the growth of the MDR strain (Figure 6I). In conclusion, no clear trend toward CR manipulation by prebiotic intervention was obtained. The diversity of changes in CR in response to prebiotic intervention is therefore likely related to intraindividual taxonomic and functional differences in the microbiota, suggesting that the ability to metabolize these specific compounds into protective factors is highly conditional on the sample's unique microbial composition, a pattern also observed for the probiotic strain MK01. In addition, prebiotic compounds often require longer-term, sustained exposure *in vivo* to significantly enrich specific beneficial taxa and induce measurable functional shifts (e.g., increased SCFA output), changes that typically take several days to fully manifest. Thus, the lack of a clear, uniform trend is likely influenced by the short period of the intervention within our 24-h *ex vivo* timeframe.

## Discussion

The composition of the intestinal microbiota has a profound effect on individual susceptibility to pathogen colonization and the severity of intestinal inflammation through both direct and indirect mechanisms (Caballero-Flores et al., 2023). Factors associated with a reduction in CR to certain members of the *Enterobacterales* order, such as *Salmonella* spp., *Citrobacter* spp., EC, or KP, include reduced overall species diversity and shifts in species evenness (Cheng et al., 2022; Honda et al., 2023; Spragge et al., 2023), lower levels of SCFA-producing bacteria (Sorbara et al., 2018; Osbelt et al., 2021), increased luminal pH (Sorbara and Pamer, 2019), and disruption of a balanced microbial community that normally provides effective niche competition (Velazquez et al., 2019; Oliveira et al., 2020; Eberl et al., 2021; Furuichi et al., 2024; Wende et al., 2025). Nevertheless, although the concept of CR has been extensively researched across various hosts, including livestock, it remains uncertain whether and how comparable principles are present in wild and farm animals, particularly given their less controlled and highly undefined microbiota states. To address this gap, our objective was to compare natural CR across different animal species, both domestic and wild, utilizing an artificial spike-in assay. Furthermore, we aimed to identify potential correlations with the taxonomic composition of the microbiome, pH, and SCFA levels, with particular emphasis on the comparisons between domestic and wild animals.

A growing body of literature indicates a negative correlation between microbiome diversity and the abundance of MDR *Enterobacterales*, suggesting that higher diversities of resident microorganisms in the microbiome are crucial for effective CR (Mayfield and Stouffer, 2017; Spragge et al., 2023). This phenomenon is often attributed to the sophisticated ecological balance among resident microorganisms, which confers enhanced stability and competitive exclusion against invading pathogens when intestinal microbiota diversity is high (Osbelt et al., 2021). However, in our *ex vivo* system, the overall diversity and relative abundance of individual microbial taxa did not significantly differ between the non-spiked and MDR EC-spiked samples. This observation suggests that, beyond mere taxonomic richness, the functional redundancy within the microbial community plays a critical role in its ability to buffer against external perturbations. Functional redundancy ensures that multiple species can perform similar essential roles or metabolic pathways, thereby maintaining ecological stability and competitive exclusion even when challenged by invading strains (Spragge et al., 2023; Wende et al., 2025). Our results emphasize the potential of direct microbial interventions to improve CR. Artificial delivery of *K. oxytoca* strain MK01, a commensal *Enterobacterales* member previously described to promote CR in humans and mice (Osbelt et al., 2021; Schluter et al., 2023; Osbelt et al., 2024), was sufficient to reduce the CFUs of MDR ECs and KPs in dairy cattle and porcine samples by 10- to 100-fold compared with those in non-probiotic-treated control samples. We acknowledge that the selected ratio used in the input may favor the probiotic, and future experiments using lower or equal inocula (e.g., 1:1) will be needed to assess competitive efficacy under more physiologically balanced conditions. Nevertheless, this remarkable effect underlines the potential of direct microbial competition to improve CR against MDR pathogens. In contrast, the prebiotic tannic acid and β-glucan-rich algal extract had only minor effects on the CR of the MDR strains in our *ex vivo* model. This discrepancy could be due to the direct, immediate competitive effect of the probiotic, as opposed to the more indirect, community-modulating effects expected from the prebiotics, which may require longer incubation times or *in vivo* conditions to reach their full potential. While the question of whether the combined feeding of prebiotic and probiotic supplements can effectively limit the proliferation of MDR *Enterobacterales* in live animals requires further *in vivo* investigation, our data provide compelling evidence that similar underlying factors determine the extent of CR in different mammalian hosts beyond humans and mice.

Comparable to the variability observed in larger human cohorts (Chen et al., 2021), we have found large interindividual variability in SCFA concentration of domestic animals and, to a lesser extent, in wildlife samples. Contrary to the general belief that fecal SCFA concentrations are usually higher in ruminants than in monogastric animals (e.g., pigs), our analysis showed a distinct trend. Our results broadly correlated with two key factors influencing SCFA values. First, we observed a significantly greater proportion of *Enterobacterales* and increased SCFA levels in pig samples compared to ruminant samples. This could be related to the diets of domestic pigs, which are typically formulated with closely adjusted protein levels and highly digestible protein sources on the farms from which we collected fecal samples. It is important to note that not all SCFAs are derived exclusively from saccharolytic fermentation. Proteolytic fermentation also contributes, especially for branched-chain SCFAs such as isovaleric acid and isobutyric acid, which are derived from amino acids such as isoleucine, leucine, and valine. Since the diet of the investigated domestic pigs was high in protein and fat, this could be a partial explanation for the comparatively higher SCFA levels in their feces (Sebastià et al., 2024). Second, our analysis revealed significantly lower concentrations of various SCFAs in wild animals (deer and wild boar) compared to their domestic counterparts. This result was rather unexpected, as a high-fiber diet characteristic of wild animals usually leads to a greater number of SCFA producers in the large intestine, whereas such a diet is often reduced in modern, westernized human populations (Claesson et al., 2012). While differences in dietary habits and intake of fermentable fiber may explain some of the observed variance between different animal hosts, definitive conclusions regarding the exact dietary components driving SCFA production remain speculative without detailed knowledge of the specific dietary composition. In addition, although our protocol aimed to preserve sample integrity, SCFA profiles can be dynamic post-defecation, influenced by ongoing microbial activity and volatility, which may impact measured concentrations. Future studies with larger cohorts would be beneficial for further confirming and expanding upon these observations. Nevertheless, it is plausible that these variations in SCFA levels contribute to the differences in CR observed between animal species, likely through mechanisms similar to those described in humans and mice (Caballero-Flores et al., 2023).

This study has several limitations. Firstly, the sample size of animal fecal samples (*n* = 82), particularly for wildlife, may be considered relatively small, thereby limiting statistical power and potentially leading to unreliable statistical differences or failure to detect subtle yet biologically relevant associations. While comparable to or exceeding that of some other studies examining fecal microbiomes of animals (e.g., Wu et al., 2021; Zhang et al., 2024, 2025), a larger cohort would provide greater statistical power and potentially reveal more subtle associations. To improve the robustness and generalizability of the data generated in this pilot study, future studies should significantly increase sample numbers, particularly for underrepresented wildlife populations. Secondly, seasonal variations in diet and environmental conditions are known to influence both the composition of the microbiota and SCFA production in wild animals (Lin et al., 2024). Although samples were collected during the winter season (between 0 °C and 10 °C) to mitigate rapid degradation, the potential influence of seasonal changes, especially dietary changes, on microbiota composition and SCFA production cannot be completely ruled out. Practical limitations, including legal restrictions (e.g., closed seasons, protection of juveniles), further restricted sampling opportunities and may have biased the timing of sampling. Future studies with larger, seasonally stratified sampling are warranted to address this limitation. Future studies should move beyond single-season snapshots to include longitudinal sampling of the same populations across different seasons or under varied dietary regimes (e.g., comparing grass-fed vs. feedlot cattle). Controlling for or accounting for these dynamic environmental variables will be essential to determine how robust the observed CR differences and intervention efficacies are under different real-world conditions. More importantly, the overall correlation between total SCFA concentration and CR is not absolute, requiring more cautious interpretation. For example, wild boar samples showed significantly lower SCFA concentrations than domestic pigs, yet provided comparable or even stronger CR against KP-ST395 and KP-ST307. This key finding, along with the inconsistent efficacy of prebiotic interventions across different hosts and strains, strongly suggests that SCFA concentrations are an incomplete proxy for CR. Instead, CR is likely governed by: (i) specific SCFA profiles, where the ratio or individual concentrations of certain SCFAs (e.g., butyrate or propionate) are more influential than the total SCFA load; or (ii) non-SCFA mechanisms, such as direct microbial antagonism (e.g., bacteriocin production or competitive utilization of non-SCFA resources). However, future research is needed to confirm these assumptions. Thirdly, while the *ex vivo* spike-in assay is a valuable tool for assessing CR, it does not fully represent the complex *in vivo* environment (Osbelt et al., 2021; Spragge et al., 2023). Critical host factors such as luminal pH gradients, host immunity, intestinal motility and mucus layer interactions, all of which can influence pathogen colonization *in vivo*, are not accounted for in this simplified, artificial assay. In addition, the initial total microbial load of the fecal extracts was not standardized across different species prior to incubation, which could have altered the metabolic profiles, spatial organization, and competitive dynamics relative to the *in vivo* state. In addition, while the four selected MDR strains represent clinically relevant high-risk clonal lineages, they do not encompass the full diversity of MDR pathogens (e.g., *Salmonella* or *Enterobacter* spp.) or even the full diversity within their respective genera. Thus, extrapolating these findings should be done with caution. Future studies will include a broader panel of MDR strains from several *Enterobacterales* species. Also, as mentioned above the co-inoculation ratio of 1:10 (pathogen:probiotic) was selected to ensure effective colonization and to reflect a preventive, rather than therapeutic, intervention scenario. Future experiments employing lower or equal inocula (e.g., 1:1) will be necessary to assess competitive efficacy under more physiologically balanced conditions. Finally, while our study employed metatranscriptomic analysis to explore gene expression within the microbial communities, the absence of shotgun metagenomic sequencing precluded a comprehensive determination of the full functional potential (i.e., the entire gene content) of the microbial communities and the precise identification of specific genes or signaling pathways that contribute to CR in domestic and wild animals. Future studies incorporating shotgun metagenomics are needed to provide a more holistic functional characterization of these complex microbial ecosystems.

In conclusion, our results show that natural variations in the gut microbiota of different animal species influence individual CR to invading MDR *Enterobacterales* strains. Furthermore, we have shown a correlation between the concentrations of different SCFAs and the abundances of *Enterobacterales* and CR. These findings align with the overall ecological concepts proposed in humans and mice, suggesting that enterobacterial species are key species conferring CR against various MDR strains (Schluter et al., 2023; Spragge et al., 2023; Furuichi et al., 2024), and further support the exploration of well-characterized enterobacterial strains, such as *K. oxytoca* strain MK01, as promising probiotic candidates for decolonizing MDR *Enterobacterales* in animals. Importantly, while the present results indicate that *K. oxytoca* MK01 can exert protective in experimental models, it is important to note that *Klebsiella* species include opportunistic pathogens. Therefore, before any translational application, extensive safety assessments and regulatory evaluations would be required to confirm the strain's suitability and safety profile for potential probiotic use.

## Data Availability

The datasets presented in this study can be found in online repositories. The names of the repository/repositories and accession number(s) can be found below: https://www.ebi.ac.uk/ena, PRJEB79754 and PRJEB80637.

## Supplementary material

Supplementary Figure S1 Cecal material from antibiotic treated mice was deliberately inoculated with varying ratios of *Klebsiella oxytoca* MK01 and *Klebsiella pneumoniae* (KPST395) (1:1 up to 100:1). Following inoculation, the samples were incubated under anaerobic conditions for 24 h, after which the Kpn MDR bacterial loads were quantified via selective agar plates. **(A)** Quantification of colony forming units (CFUs) per ml of cecum content for Kpn ST395 in control and samples spiked with MK01 in various ratios. **(B)** Log-fold change in comparison to CFU amount in untreated control sample. Results represent one biological experiment performed in triplicates.

## References

[B1] Antimicrobial Resistance Collaborators (2022). Global burden of bacterial antimicrobial resistance in 2019: a systematic analysis. Lancet 399, 629–655. doi: 10.1016/S0140-6736(21)02724-0 37500506

[B2] Atarashi K. Tanoue T. Ando M. Kamada N. Nagano Y. Narushima S. . (2015). Th17 cell induction by adhesion of microbes to intestinal epithelial cells. Cell 163, 367–380. doi: 10.1016/j.cell.2015.08.058 26411289 PMC4765954

[B3] Belkaid Y. Hand T. W. (2014). Role of the microbiota in immunity and inflammation. Cell 157, 121–141. doi: 10.1016/j.cell.2014.03.011 24679531 PMC4056765

[B4] Brendecke J. Homeier-Bachmann T. Schmitz Ornés A. Guenther S. Heiden S. E. Schwabe M. . (2022). Multidrug-resistant high-risk Escherichia coli and Klebsiella pneumoniae clonal lineages occur in black-headed gulls from two conservation islands in Germany. Antibiotics 11:1357. doi: 10.3390/antibiotics11101357 36290018 PMC9598702

[B5] Caballero-Flores G. Pickard J. M. Núñez G. (2023). Microbiota-mediated colonization resistance: mechanisms and regulation. Nat. Rev. Microbiol. 21, 347–360. doi: 10.1038/s41579-022-00833-7 36539611 PMC10249723

[B6] Cantalapiedra C. P. Hernández-Plaza A. Letunic I. Bork P. Huerta-Cepas J. (2021). eggNOG-mapper v2: functional annotation, orthology assignments, and domain prediction at the metagenomic scale. Mol. Biol. Evol. 38, 5825–5829. doi: 10.1093/molbev/msab293 34597405 PMC8662613

[B7] Caporaso J. G. Kuczynski J. Stombaugh J. Bittinger K. Bushman F. D. Costello E. K. . (2010). QIIME allows analysis of high-throughput community sequencing data. Nat. Methods 7, 335–336. doi: 10.1038/nmeth.f.303 20383131 PMC3156573

[B8] Caporaso J. G. Lauber C. L. Walters W. A. Berg-Lyons D. Lozupone C. A. Turnbaugh P. J. . (2011). Global patterns of 16S rRNA diversity at a depth of millions of sequences per sample. Proc. Natl. Acad. Sci. U. S. A. 108, 4516–4522. doi: 10.1073/pnas.1000080107 20534432 PMC3063599

[B9] Chen L. Wang D. Garmaeva S. Kurilshikov A. Vich Vila A. Gacesa R. . (2021). The long-term genetic stability and individual specificity of the human gut microbiome. Cell 184, 2302–2315.e2312. doi: 10.1016/j.cell.2021.03.024 33838112

[B10] Cheng A. G. Ho P.-Y. Aranda-Díaz A. Jain S. Yu F. B. Meng X. . (2022). Design, construction, and in vivo augmentation of a complex gut microbiome. Cell 185, 3617–3636.e3619. doi: 10.1016/j.cell.2022.08.003 36070752 PMC9691261

[B11] Choi J. Yadav S. Vaddu S. Thippareddi H. Kim W. K. (2023). In vitro and in vivo evaluation of tannic acid as an antibacterial agent in broilers infected with Salmonella Typhimurium. Poult. Sci. 102:102987. doi: 10.1016/j.psj.2023.102987 37844525 PMC10585643

[B12] Claesson M. J. Jeffery I. B. Conde S. Power S. E. O'Connor E. M. Cusack S. . (2012). Gut microbiota composition correlates with diet and health in the elderly. Nature 488, 178–184. doi: 10.1038/nature11319 22797518

[B13] Eberl C. Weiss A. S. Jochum L. M. Durai Raj A. C. Ring D. Hussain S. . (2021). E. coli enhance colonization resistance against Salmonella Typhimurium by competing for galactitol, a context-dependent limiting carbon source. Cell Host Microbe 29, 1680–1692.e1687. doi: 10.1016/j.chom.2021.09.004 34610296

[B14] Edgar R. C. (2010). Search and clustering orders of magnitude faster than BLAST. Bioinformatics 26, 2460–2461. doi: 10.1093/bioinformatics/btq461 20709691

[B15] Eger E. Domke M. Heiden S. E. Paditz M. Balau V. Huxdorff C. . (2022). Highly virulent and multidrug-resistant Escherichia coli sequence type 58 from a sausage in Germany. Antibiotics 11:1006. doi: 10.3390/antibiotics11081006 35892394 PMC9331442

[B16] Eger E. Homeier-Bachmann T. Adade E. Dreyer S. Heiden S. E. Lübcke S. . (2024). Carbapenem- and cefiderocol-resistant Enterobacterales in surface water in Kumasi, Ashanti Region, Ghana. JAC-Antimicrob. Resist. 6:dlae021. doi: 10.1093/jacamr/dlae021 38449514 PMC10915899

[B17] Fang K. Jin X. Hong S. H. (2018). Probiotic Escherichia coli inhibits biofilm formation of pathogenic E. coli via extracellular activity of DegP. Sci. Rep. 8:4939. doi: 10.1038/s41598-018-23180-1 29563542 PMC5862908

[B18] Fischbach M. A. Sonnenburg J. L. (2011). Eating for two: how metabolism establishes interspecies interactions in the gut. Cell Host Microbe 10, 336–347. doi: 10.1016/j.chom.2011.10.002 22018234 PMC3225337

[B19] Furuichi M. Kawaguchi T. Pust M.-M. Yasuma-Mitobe K. Plichta D. R. Hasegawa N. . (2024). Commensal consortia decolonize Enterobacteriaceae via ecological control. Nature 633, 878–886. doi: 10.1038/s41586-024-07960-6 39294375 PMC11424487

[B20] Hofmann J. D. Otto A. Berges M. Berges M. Biedendieck R. Michel A.-M. . (2018). Metabolic reprogramming of Clostridioides difficile during the stationary phase with the induction of toxin production. Front. Microbiol. 9:1970. doi: 10.3389/fmicb.2018.01970 30186274 PMC6110889

[B21] Homeier-Bachmann T. Heiden S. E. Lübcke P. K. Bachmann L. Bohnert J. A. Zimmermann D. . (2021). Antibiotic-resistant Enterobacteriaceae in wastewater of abattoirs. Antibiotics 10:568. doi: 10.3390/antibiotics10050568 34065908 PMC8150771

[B22] Honda K. Furuichi M. Kawaguchi T. Pust M. M. Yasuma-Mitobe K. Plichta D. . (2023). Rationally-defined microbial consortia suppress multidrug-resistant proinflammatory Enterobacteriaceae via ecological control. Research Square. 23:rs.3.rs-3462622. doi: 10.21203/rs.3.rs-3462622/v1 37961431 PMC10635318

[B23] Ivanov I. I. Atarashi K. Manel N. Brodie E. L. Shima T. Karaoz U. . (2009). Induction of intestinal Th17 cells by segmented filamentous bacteria. Cell 139, 485–498. doi: 10.1016/j.cell.2009.09.033 19836068 PMC2796826

[B24] Jacobson A. Lam L. Rajendram M. Tamburini F. Honeycutt J. Pham T. . (2018). A gut commensal-produced metabolite mediates colonization resistance to Salmonella infection. Cell Host Microbe 24, 296–307.e297. doi: 10.1016/j.chom.2018.07.002 30057174 PMC6223613

[B25] Jaramillo-Jaramillo A. S. McClure J. T. Stryhn H. Tahlan K. Sanchez J. (2024). Effects of storage conditions on the microbiota of fecal samples collected from dairy cattle. PLoS ONE 19:e0308571. doi: 10.1371/journal.pone.0308571 39121104 PMC11315314

[B26] Kamada N. Kim Y.-G. Sham H. P. Vallance B. A. Puente J. L. Martens E. C. . (2012). Regulated virulence controls the ability of a pathogen to compete with the gut microbiota. Science 336, 1325–1329. doi: 10.1126/science.1222195 22582016 PMC3439148

[B27] Lam L. H. Monack D. M. (2014). Intraspecies competition for niches in the distal gut dictate transmission during persistent Salmonella infection. PLoS Pathog. 10:e1004527. doi: 10.1371/journal.ppat.1004527 25474319 PMC4256465

[B28] Langmead B. Wilks C. Antonescu V. Charles R. (2018). Scaling read aligners to hundreds of threads on general-purpose processors. Bioinformatics 35, 421–432. doi: 10.1093/bioinformatics/bty648 30020410 PMC6361242

[B29] Liao Y. Smyth G. K. Shi W. (2013). featureCounts: an efficient general purpose program for assigning sequence reads to genomic features. Bioinformatics 30, 923–930. doi: 10.1093/bioinformatics/btt656 24227677

[B30] Lin L. Guo Z. Bai J. Zhong Z. Li J. Guo Q. (2024). Seasonal covariations of diet-gut microbiota in the adaptation of the newly reintroduced Père David's deer (Elaphurus davidianus) to the northern habitat. Global Ecol. Conserv. 52:e02983. doi: 10.1016/j.gecco.2024.e02983

[B31] Love M. I. Huber W. Anders S. (2014). Moderated estimation of fold change and dispersion for RNA-seq data with DESeq2. Genome Biol. 15:550. doi: 10.1186/s13059-014-0550-8 25516281 PMC4302049

[B32] Lu J. Rincon N. Wood D. E. Breitwieser F. P. Pockrandt C. Langmead B. . (2022). Metagenome analysis using the Kraken software suite. Nat. Protoc. 17, 2815–2839. doi: 10.1038/s41596-022-00738-y 36171387 PMC9725748

[B33] Mayfield M. M. Stouffer D. B. (2017). Higher-order interactions capture unexplained complexity in diverse communities. Nat. Ecol. Evol. 1:0062. doi: 10.1038/s41559-016-0062 28812740

[B34] McMurdie P. J. Holmes S. (2013). phyloseq: an R package for reproducible interactive analysis and graphics of microbiome census data. PLoS ONE 8:e61217. doi: 10.1371/journal.pone.0061217 23630581 PMC3632530

[B35] Neumann-Schaal M. Hofmann J. D. Will S. E. Schomburg D. (2015). Time-resolved amino acid uptake of Clostridium difficile 630Δerm and concomitant fermentation product and toxin formation. BMC Microbiol. 15:281. doi: 10.1186/s12866-015-0614-2 26680234 PMC4683695

[B36] Ng K. M. Ferreyra J. A. Higginbottom S. K. Lynch J. B. Kashyap P. C. Gopinath S. . (2013). Microbiota-liberated host sugars facilitate post-antibiotic expansion of enteric pathogens. Nature 502, 96–99. doi: 10.1038/nature12503 23995682 PMC3825626

[B37] Oliveira R. A. Ng K. M. Correia M. B. Cabral V. Shi H. Sonnenburg J. L. . (2020). Klebsiella michiganensis transmission enhances resistance to Enterobacteriaceae gut invasion by nutrition competition. Nat. Microbiol. 5, 630–641. doi: 10.1038/s41564-019-0658-4 31959968

[B38] Osbelt L. Almási É. d. H. Wende M. Kienesberger S. Voltz A. Lesker T. R. . (2024). Klebsiella oxytoca inhibits Salmonella infection through multiple microbiota-context-dependent mechanisms. Nat. Microbiol. 9, 1792–1811. doi: 10.1038/s41564-024-01710-0 38862602 PMC11222139

[B39] Osbelt L. Thiemann S. Smit N. Lesker T. R. Schröter M. Gálvez E. J. C. . (2020). Variations in microbiota composition of laboratory mice influence Citrobacter rodentium infection via variable short-chain fatty acid production. PLoS Pathog. 16:e1008448. doi: 10.1371/journal.ppat.1008448 32208465 PMC7141690

[B40] Osbelt L. Wende M. Almási É. Derksen E. Muthukumarasamy U. Lesker T. R. . (2021). Klebsiella oxytoca causes colonization resistance against multidrug-resistant K. pneumoniae in the gut via cooperative carbohydrate competition. Cell Host Microbe 29, 1663–1679.e1667. doi: 10.1016/j.chom.2021.09.003 34610293

[B41] Peng Z. Hu Z. Li Z. Zhang X. Jia C. Li T. . (2022). Antimicrobial resistance and population genomics of multidrug-resistant Escherichia coli in pig farms in mainland China. Nat. Commun. 13:1116. doi: 10.1038/s41467-022-28750-6 35236849 PMC8891348

[B42] Plaza-Rodríguez C. Alt K. Grobbel M. Hammerl J. A. Irrgang A. Szabo I. . (2021). Wildlife as sentinels of antimicrobial resistance in Germany? Front. Vet. Sci. 7:627821. doi: 10.3389/fvets.2020.627821 33585611 PMC7873465

[B43] Price M. N. Dehal P. S. Arkin A. P. (2010). FastTree 2 – approximately maximum-likelihood trees for large alignments. PLoS ONE 5:e9490. doi: 10.1371/journal.pone.0009490 20224823 PMC2835736

[B44] Quast C. Pruesse E. Yilmaz P. Gerken J. Schweer T. Yarza P. . (2012). The SILVA ribosomal RNA gene database project: improved data processing and web-based tools. Nucleic Acids Res. 41, D590–D596. doi: 10.1093/nar/gks1219 23193283 PMC3531112

[B45] Schaufler K. Nowak K. Düx A. Semmler T. Villa L. Kourouma L. . (2018). Clinically relevant ESBL-producing K. pneumoniae ST307 and E. coli ST38 in an urban West African rat population. Front. Microbiol. 9:150. doi: 10.3389/fmicb.2018.00150 29479341 PMC5812336

[B46] Schaufler K. Wieler L. H. Semmler T. Ewers C. Guenther S. (2013). ESBL-plasmids carrying toxin-antitoxin systems can be “cured” of wild-type Escherichia coli using a heat technique. Gut Pathog. 5:34. doi: 10.1186/1757-4749-5-34 24245987 PMC4177129

[B47] Schluter J. Djukovic A. Taylor B. P. Yan J. Duan C. Hussey G. A. . (2023). The TaxUMAP atlas: efficient display of large clinical microbiome data reveals ecological competition in protection against bacteremia. Cell Host Microbe 31, 1126–1139.e1126. doi: 10.1016/j.chom.2023.05.027 37329880 PMC10527165

[B48] Schulze C. Stamer L. L. M. Huss S. K. Schaufler K. Guenther S. Schultze N. (2021). Establishment of a quantification method for β-glucans and their immune activity potential for quality control of β-glucan containing products. Carbohydr. Res. 504:108327. doi: 10.1016/j.carres.2021.108327 33934035

[B49] Sebastià C. Folch J. M. Ballester M. Estellé J. Passols M. Muñoz M. . (2024). Interrelation between gut microbiota, SCFA, and fatty acid composition in pigs. mSystems 9:e01049–01023. doi: 10.1128/msystems.01049-23 38095419 PMC10804976

[B50] Seemann T. (2014). Prokka: rapid prokaryotic genome annotation. Bioinformatics 30, 2068–2069. doi: 10.1093/bioinformatics/btu153 24642063

[B51] Sorbara M. T. Dubin K. Littmann E. R. Moody T. U. Fontana E. Seok R. . (2018). Inhibiting antibiotic-resistant Enterobacteriaceae by microbiota-mediated intracellular acidification. J. Exp. Med. 216, 84–98. doi: 10.1084/jem.20181639 30563917 PMC6314524

[B52] Sorbara M. T. Pamer E. G. (2019). Interbacterial mechanisms of colonization resistance and the strategies pathogens use to overcome them. Mucosal Immunol. 12, 1–9. doi: 10.1038/s41385-018-0053-0 29988120 PMC6312114

[B53] Spragge F. Bakkeren E. Jahn M. T. B. N. Araujo E. Pearson C. F. Wang X. . (2023). Microbiome diversity protects against pathogens by nutrient blocking. Science 382:eadj3502. doi: 10.1126/science.adj3502 38096285 PMC7616675

[B54] Velazquez E. M. Nguyen H. Heasley K. T. Saechao C. H. Gil L. M. Rogers A. W. L. . (2019). Endogenous Enterobacteriaceae underlie variation in susceptibility to Salmonella infection. Nat. Microbiol. 4, 1057–1064. doi: 10.1038/s41564-019-0407-8 30911125 PMC6533147

[B55] Wang Q. Garrity G. M. Tiedje J. M. Cole J. R. (2007). Naïve Bayesian classifier for rapid assignment of rRNA sequences into the new bacterial taxonomy. Appl. Environ. Microbiol. 73, 5261–5267. doi: 10.1128/AEM.00062-07 17586664 PMC1950982

[B56] Wende M. Osbelt L. Eisenhard L. Lesker T. R. Damaris B. F. Mutukumarasamy U. . (2025). Suppression of gut colonization by multidrug-resistant Escherichia coli clinical isolates through cooperative niche exclusion. Nat. Commun. 16:5426. doi: 10.1038/s41467-025-61327-7 40593813 PMC12215308

[B57] Wu K. Xu Y. Zhang W. Mao H. Chen B. Zheng Y. . (2021). Differences in fecal microbiome and antimicrobial resistance between captive and free-range sika deer under the same exposure of antibiotic anthelmintics. Microbiol. Spectr. 9:e01918–01921. doi: 10.1128/Spectrum.01918-21 34851181 PMC8635127

[B58] Zhang C. Yu Y. Yue L. Chen Y. Chen Y. Liu Y. . (2025). Gut microbiota profiles of sympatric snub-nosed monkeys and macaques in Qinghai-Tibetan Plateau show influence of phylogeny over diet. Commun. Biol. 8:95. doi: 10.1038/s42003-025-07538-6 39833341 PMC11747120

[B59] Zhang J. Song P. Jiang F. Zhang T. (2024). Exploring the population interaction of Przewalski's gazelle (Procapra przewalskii) based on the variations in gut microbiota across diverse geographic populations. Front. Microbiol. 15:1439554. doi: 10.3389/fmicb.2024.1439554 39234536 PMC11371741

[B60] Zmora N. Zilberman-Schapira G. Suez J. Mor U. Dori-Bachash M. Bashiardes S. . (2018). Personalized gut mucosal colonization resistance to empiric probiotics is associated with unique host and microbiome features. Cell 174, 1388–1405.e1321. doi: 10.1016/j.cell.2018.08.041 30193112

