# Supplementary figures and images for: A comparative ex vivo evaluation of colonization resistance against multidrug-resistant Enterobacterales in fecal samples from various animal species and strategies to augment it
Source: Front Microbiol. 2026 Jan 26;16:1695208. doi: 10.3389/fmicb.2025.1695208 (PMC12883652; doi:10.3389/fmicb.2025.1695208)

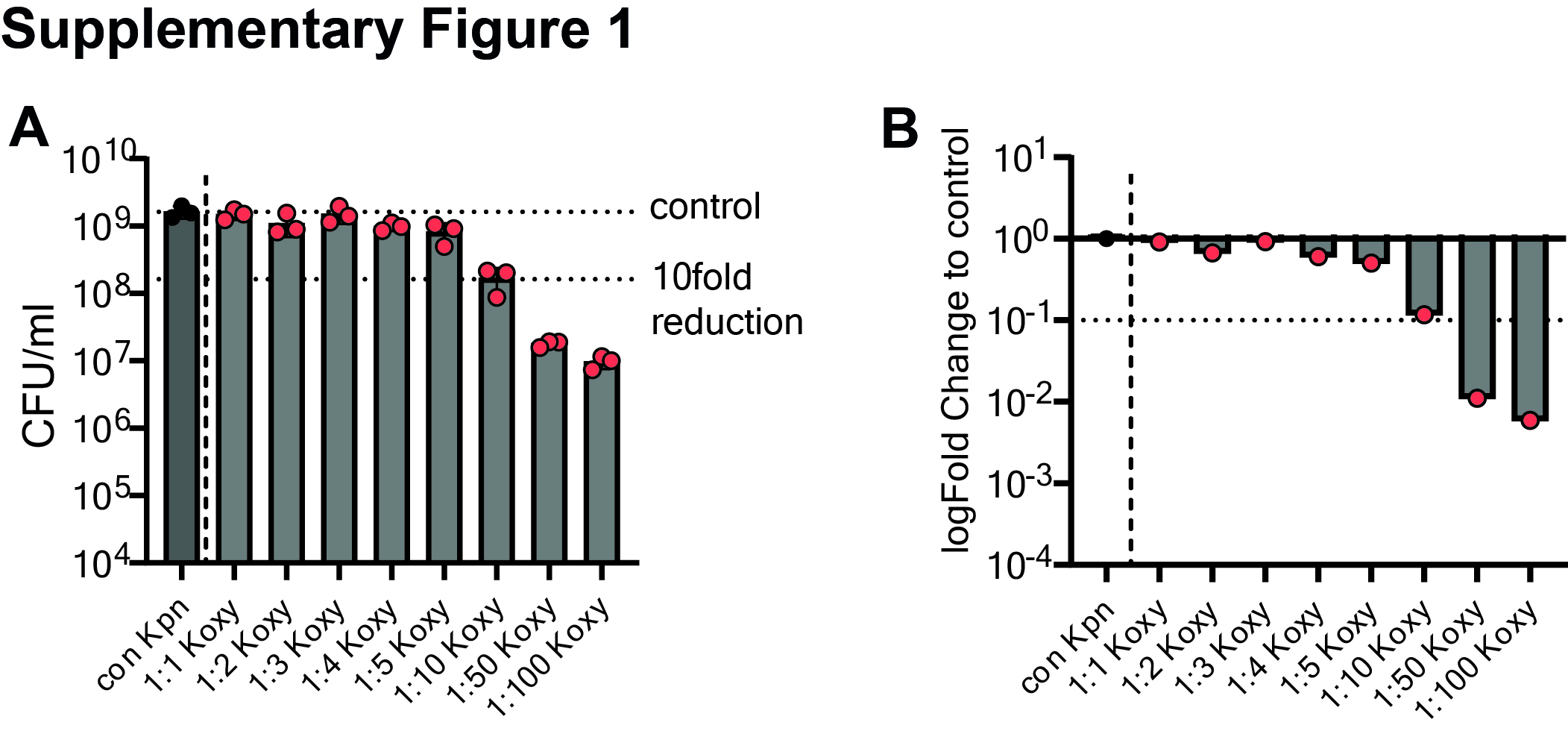

Supplement: Supplementary Figure S1 — Cecal material from antibiotic treated mice was deliberately inoculated with varying ratios of Klebsiella oxytoca MK01 and Klebsiella pneumoniae (KPST395) (1:1 up to 100:1). Following inoculation, the samples were incubated under anaerobic conditions for 24 h, after which the Kpn MDR bacterial loads were quantified via selective agar plates. (A) Quantification of colony forming units (CFUs) per ml of cecum content for Kpn ST395 in control and samples spiked with MK01 in various ratios. (B) Log-fold change in comparison to CFU amount in untreated control sample. Results represent one biological experiment performed in triplicates. [file Image_1.jpg]
